# Contractility measurements for cardiotoxicity screening with ventricular myocardial slices of pigs

**DOI:** 10.1093/cvr/cvad141

**Published:** 2023-11-02

**Authors:** Runzhu Shi, Marius Reichardt, Dominik J Fiegle, Linda K Küpfer, Titus Czajka, Zhengwu Sun, Tim Salditt, Andreas Dendorfer, Thomas Seidel, Tobias Bruegmann

**Affiliations:** Institute for Cardiovascular Physiology, University Medical Center Göttingen, Humboldtallee 23, 37073 Göttingen, Göttingen, Germany; International Research Training Group 1816, University Medical Center Göttingen, Göttingen, Germany; Institute for Cardiovascular Physiology, University Medical Center Göttingen, Humboldtallee 23, 37073 Göttingen, Göttingen, Germany; Institute for X-ray Physics, University of Göttingen, Göttingen, Germany; Institute of Cellular and Molecular Physiology, Friedrich-Alexander-University Erlangen-Nürnberg, Erlangen, Germany; Institute of Cellular and Molecular Physiology, Friedrich-Alexander-University Erlangen-Nürnberg, Erlangen, Germany; Institute for X-ray Physics, University of Göttingen, Göttingen, Germany; Walter-Brendel-Centre of Experimental Medicine, Hospital of the University Munich, Munich, Germany; Institute for X-ray Physics, University of Göttingen, Göttingen, Germany; Cluster of Excellence ‘Multiscale Bioimaging: from Molecular Machines to Networks of Excitable Cells’ (MBExC), University of Göttingen, Göttingen, Germany; Walter-Brendel-Centre of Experimental Medicine, Hospital of the University Munich, Munich, Germany; German Centre of Cardiovascular Research (DZHK), Munich Heart Alliance, Munich, Germany; Institute of Cellular and Molecular Physiology, Friedrich-Alexander-University Erlangen-Nürnberg, Erlangen, Germany; Institute for Cardiovascular Physiology, University Medical Center Göttingen, Humboldtallee 23, 37073 Göttingen, Göttingen, Germany; Cluster of Excellence ‘Multiscale Bioimaging: from Molecular Machines to Networks of Excitable Cells’ (MBExC), University of Göttingen, Göttingen, Germany; German Center for Cardiovascular Research (DZHK), Partner site Göttingen, Göttingen, Germany

**Keywords:** Cardiotoxicity, Ventricular slices, Screening, Drug-induced long QT syndrome, Cardiac arrhythmia

## Abstract

**Aims:**

Cardiotoxicity is one major reason why drugs do not enter or are withdrawn from the market. Thus, approaches are required to predict cardiotoxicity with high specificity and sensitivity. Ideally, such methods should be performed within intact cardiac tissue with high relevance for humans and detect acute and chronic side effects on electrophysiological behaviour, contractility, and tissue structure in an unbiased manner. Herein, we evaluate healthy pig myocardial slices and biomimetic cultivation setups (BMCS) as a new cardiotoxicity screening approach.

**Methods and results:**

Pig left ventricular samples were cut into slices and spanned into BMCS with continuous electrical pacing and online force recording. Automated stimulation protocols were established to determine the force–frequency relationship (FFR), frequency dependence of contraction duration, effective refractory period (ERP), and pacing threshold. Slices generated 1.3 ± 0.14 mN/mm^2^ force at 0.5 Hz electrical pacing and showed a positive FFR and a shortening of contraction duration with increasing pacing rates. Approximately 62% of slices were able to contract for at least 6 days while showing stable ERP, contraction duration–frequency relationship, and preserved cardiac structure confirmed by confocal imaging and X-ray diffraction analysis. We used specific blockers of the most important cardiac ion channels to determine which analysis parameters are influenced. To validate our approach, we tested five drug candidates selected from the Comprehensive *in vitro* Proarrhythmia Assay list as well as acetylsalicylic acid and DMSO as controls in a blinded manner in three independent laboratories. We were able to detect all arrhythmic drugs and their respective mode of action on cardiac tissue including inhibition of Na^+^, Ca^2+^, and hERG channels as well as Na^+^/Ca^2+^ exchanger.

**Conclusion:**

We systematically evaluate this approach for cardiotoxicity screening, which is of high relevance for humans and can be upscaled to medium-throughput screening. Thus, our approach will improve the predictive value and efficiency of preclinical cardiotoxicity screening.

**Time of primary review: 40 days**

## 1. Introduction

Cardiac side effects of drugs can cause lethal arrhythmias, alter contractility, and induce heart failure^1^ and are one major reason for drug failure along different stages of drug development^2^ and withdrawals from the market.^3^ Thus, efficient preclinical screening of newly developed drugs and therapeutic approaches for potential side effects on the heart—cardiotoxicity screening—is crucial not only to detect unsafe compounds to reduce the risk of participants in clinical studies and patients but also to prevent false withdrawals and economic losses.^2^ Most frequently, drugs like anti-epileptics, anti-depressants, and antibiotics^4–6^ act directly on cardiomyocytes and block the human ether-a-go-go-related gene potassium channel (hERG K^+^ channel), which delays the cardiac repolarization resulting in prolonged action potential duration (APD) and QT duration within the electrocardiogram (ECG).^7^ This so-called drug-induced long QT syndrome can lead to early after depolarizations and potentially fatal arrhythmia, e.g. torsades de pointes or ventricular fibrillation.^6,8,9^ However, side effects on all other cardiac ion channels and alterations of the electrophysiological homeostasis can be proarrhythmic as well.^6^ For example, the short QT syndrome can be caused by blocking Ca^2+^ channels, while the Brugada syndrome can result from inhibiting Na^+^ channels.^2,10,11^ Furthermore, substances used for cancer treatment, from conventional cytotoxic agents to newer targeted and immune-based therapies such as kinase inhibitors, can lead to left ventricular dysfunction, congestive heart failure, myocardial ischaemia, and myocarditis.^1^ Such effects do not have to solely act on cardiomyocytes since, among others, doxorubicin can affect fibroblasts and thereby the heart function.^12^ Thus, ideal cardiotoxicity screening would (i) allow drug testing within intact cardiac tissue with the cell composition as close as possible to the human heart, (ii) enable to detect possible side effects on electrophysiological behaviour, contractility, and tissue structure and composition in an unbiased manner and over prolonged time periods,^13^ while (iii) minimizing the number and suffering of animals.

Still, the patch-clamp technique is the gold standard for investigating electrophysiological properties of ion channels,^14^ and for many years, cardiotoxicity assessment was solely performed on human embryonic kidney cells (HEK293 cells) overexpressing the hERG K^+^ channels.^15^ However, the artificial expression of single cardiac ion channels in genetically modified HEK293 cell lines cannot accurately model pertinent genetic, cellular, or biochemical characteristics of the human heart.^16,17^ In addition, effects on other ion channels are neglected by single ion channel screenings, which could add on or neutralize each other.^18^ Even if drug effects recorded on cardiac ion channels expressed in simple cell systems are integrated *in silico* in mathematical models of human ventricular myocytes,^19^ influences by the specific cellular environment such as regulation by second messengers and compartmentalization as well as interactions between different ion channel types will not be detected. For example, screening only for side effects on hERG channels in HEK293 cells, at least 60% of drugs would show some effects despite an otherwise rather safe side effects profile^20^ and would have to be falsely withdrawn from the route into clinical use.

Human-induced pluripotent stem cell–derived cardiomyocytes (hiPSC-CMs) provide new opportunities to create *in vitro* models of healthy and diseased human cardiomyocytes with the advantage of patient-specific disease models for precision medicine.^21^ Human-engineered heart muscles are generated from hiPSC-CMs and cardiac fibroblasts to form cardiac tissue–like structures and have been shown to enhance maturation and force development^22–24^ but are still rather immature compared to adult hearts.^25–27^

To investigate adult cardiomyocytes and hearts, isolated cardiomyocytes and intact animal hearts are widely used for cardiotoxicity screening.^2^ However, both are limited to time periods of hours.^28,29^ Furthermore, the animal models allowing investigations with larger numbers, such as mice, rats, and rabbits, show different features compared to human hearts, such as composition of ion channels.^30,31^

To bridge this gap between relevance and feasibility, myocardial slices with intact multicellular composition and cardiac structure have been explored in acute experiments since decades^32–36^ but only recently, biomimetic cultivation setups (BMCS) have been developed to enable cultivation and online contraction monitoring of ventricular slices from human failing hearts for weeks with precise control of pacing rate, pre- and afterload.^37–40^ Unfortunately, the availability of human tissue is very limited and restricted almost exclusively to failing human hearts. Since the pig heart has very similar features compared to human hearts,^31^ pig myocardial slices could represent a valuable model for cardiotoxicity screening enabling medium-throughput experiments with high relevance for humans. However, pig ventricular slices and online contraction measurements have not been explored and characterized as tool for cardiotoxicity screening.

Therefore, we evaluate in this project acute measurements and long-term cultivation of pig myocardial slices in BMCS and explore different stimulation protocols for electrophysiological characterization of drug effects and prediction of involved ion channels. To validate our experiment and analysis pipeline, we tested five drugs from the Comprehensive *in vitro* Proarrhythmia Assay (CiPA) list^41^ with known risk and mechanisms of action.

## 2. Methods

### 2.1 Tissue slicing and culture

All animal work conformed to the European Guideline for animal experiments 2010/63/EU. Pigs were killed by 40 mg/kg body weight pentobarbital and high KCl injections. Hearts were explanted and placed in ice-cold cardioplegia solution^42^ comprising 5.5 mM glucose, 0.5 mM Mg_2_SO_4_, 24 mM KCl, 20 mM NaHCO_3_, 109 mM NaCl, 0.9 mM sodium phosphate monobasic monohydrate, and 1.8 mM CaCl_2_. The middle of the free left ventricular wall was cut into ∼10 mm × 10 mm × 5 mm big blocks and embedded in 4% low-melting agarose (Agarose BioReagent, A9539, Sigma-Aldrich, USA). Following an approach described recently,^38,43,44^ slices of 300 µm thickness were cut with a vibratome (LEICA VT1200s or LEICA VT1000s, Germany) using 1.5 mm amplitude, 85/80 Hz frequency, and 0.2 mm/s speed in ice-cold slicing buffer^42^ comprising 30 mM 2,3-butanedione monoxime, 1.0 mM glucose, 10 mM HEPES, 6 mM KCl, 140 mM NaCl, 1.0 mM MgCl_2_, and 1.8 mM CaCl_2_ at pH at 7.40. Squares of 0.5 × 0.5 cm^2^ from the midmyocardium with macroscopically visible longitudinal orientation of the myofibers were selected, cut from the slices, and glued with histoacryl glue (B. Braun, Germany) on two plastic triangles, placed into MyoDish BMCS chambers (InVitroSys, Germany) filled with 4 mL Medium-199 (11150059, Life Technologies, USA) supplemented with 3% penicillin–streptomycin (15140122, Life Technologies, USA), 50 µM of the anti-oxidant β-mercaptoethanol (A11080100, Th. Geyer, Germany), 20 nM cortisol solution (C-106-1 mL, Sigma, USA), and 0.001% Ins.-Trans.-Selenium-X Suppl. 100X (51500056, Life Technologies, USA), and placed in the standard incubator with 37°C and 5% CO_2_. Electrical pacing (0.5 Hz, 80 mA, 3 ms) was performed and controlled by the MyoDish software, and contraction force was continuously recorded. After 30 min equilibration, the pre-load of the slices was re-adjusted to 1.5 mN. The medium exchange was performed with reduced penicillin–streptomycin concentration (1%) from the first day on.

### 2.2 Determination of screening parameters

Multiple contractility tests were performed daily, starting at Day 1, and during drug screening experiments, including assessment of the effective refractory period (ERP), pacing threshold, and frequency effects by combining electrical pacing with force measurements. The regular pacing rate was 0.5 Hz (biphasic, 3 ms) during long-term incubation and between series of automatic stimulation protocols. The electrical current was set to 80 mA and only lowered for testing of the pacing threshold. ERP was tested by seven regular stimuli with an interpulse interval of 2000 ms (S1) followed by an additional stimulation (S2). The interval between the last S1 and the S2 stimuli was decreased from 1320 to 100 ms by 20 ms in each step. The longest interval that failed to induce two individual contractions was defined as the ERP. To assess the stimulation threshold, stimulus current was decreased every 20 s by 2 mA, starting at 80 mA. Successful pacing was defined when the last eight stimuli of each period were 1:1 followed by a contraction, and the minimal pacing current that the tissue could follow was defined as the pacing threshold. To determine the effects of different contraction frequencies, the slices were paced with 0.1, 0.3, 0.7, 1, 1.25, 1.5, 2, and 3 for 1 min each, and 4 Hz for ~25 s. To quantify contraction force at different pacing frequencies, we selected the last 6–15 regular contractions at the end of each frequency step and analysed the force amplitude and contraction duration by the peak analysis of the LabChart8 software (ADInstruments, Sydney, Australia). The contraction force was defined as the height of the peak minus the baseline and was normalized to the nominal slice thickness of 300 µm and width of 5 mm corresponding to the width of plastic triangles. Contraction duration was defined as the time interval from the first to the last crossing of 30% of the peak height, FFR, by calculating the ratio of the force amplitude at 1.5 Hz for CiPA tests or 1.25 Hz for all others and the force at 0.3 Hz. The contraction duration–frequency relationship (CDFR) was assessed by dividing the contraction duration at 2 Hz by its value at 0.3 Hz. FFR and CDFR values above 1 indicate an increase in force and contraction duration with increasing frequencies and were thus defined as being positive and values <1 as negative. To analyze contraction parameters during long-term incubation, 15–20 peaks at regular pacing were averaged every 24 h.

### 2.3 Drug tests

We used defined blockers or activators of Na^+^ channels (lidocaine/ranolazine), Ca^2+^ channel (Bay K8644/nifedipine), hERG K^+^ channel (dofetilide/moxifloxacin/sotalol), *I*_Ks_ (JNJ 303), and Na^+^/K^+^ ATPase (ouabain) to validate which cardiac ion channels influence which analysis parameters and isoprenaline as β-adrenergic receptor agonist. Drugs were added at the indicated concentration to the BMCS from the following stocks: lidocaine (Sigma-Aldrich, USA) 100 mM dissolved in EtOH; Bay K8644 (Tocris Bioscience, UK) 300 mM dissolved in DMSO; nifedipine (Sigma-Aldrich, USA) 10 mM dissolved in DMSO; dofetilide (Sigma-Aldrich, USA) 30 mM dissolved in DMSO; sotalol hydrochloride (Sigma-Aldrich, USA) 65 mM dissolved in H_2_O; JNJ 303 25 mM dissolved in DMSO; ouabain (Sigma-Aldrich, USA) 100 mM dissolved in DMSO; moxifloxacin (Tocris Bioscience, UK) 10 mM dissolved in H_2_O; ranolazine (Selleckchem, USA) 50 mM dissolved in DMSO; and isoprenaline hydrochloride (Sigma-Aldrich, USA): 10 mM dissolved in H_2_O. These experiments were performed within 24 h after slicing, and the acute drug test experiments started 30–60 min after readjusting the pre-load. First, the ERP, pacing threshold, and frequency effects were tested to obtain baseline values. Then the respective drugs were added with increasing concentrations. After 15 min incubation for each concentration, the above described tests were performed, and the normalized values compared to time-matched controls. To detect late Na^+^ currents, slices were incubated for 3–5 days. The test protocol was performed before adding the *I*_Kr_ blockers as well as 15 min and 12 h afterwards by adding 10 μM ranolazine to confirm the presence of the late Na^+^ current.

To validate our approach, we chose five drugs from the CiPA list with various risks and mechanisms of action. Phosphate buffered saline (PBS) was used in time-matched controls, as well as the solvent DMSO and acetylsalicylic acid as a drug with no known cardiac side effects as negative controls. After the left ventricles were obtained, part of the tissue was placed into an ice-cold transport solution^38,43^ (comprising 10 mM glucose, 1 mM MgCl_2_, 24 mM KCl, 136 mM NaCl, 0.33 mM Na^+^ phosphate monobasic monohydrate, and 0.9 mM CaCl_2_) and transported together with the anonymized drugs to the two other laboratories (Institute of Cellular and Molecular Physiology, Friedrich-Alexander-University Erlangen–Nuremberg, Germany, and Walter-Brendel-Centre of Experimental Medicine, Munich, Germany). The slices were cut 24–36 h after explantation, and experiments and analyses were performed by persons blinded against the drugs on the next day. Drugs from the CiPA list were added at five different concentration steps into 3–4 mL from the following stocks: bepridil hydrochloride (Tocris Bioscience, UK) 10 mM dissolved in DMSO; ibutilide hemifumarate (Sigma-Aldrich, USA) 10 μM dissolved in H_2_O; disopyramide phosphate (Sigma-Aldrich, USA) 10 mM dissolved in H_2_O; cisapride monohydrate 100 μM dissolved in DMSO; risperidone (Sigma-Aldrich, USA) 1 mM dissolved in DMSO; and acetylsalicylic acid (Sigma-Aldrich, USA) 100 μM dissolved in H_2_O.

### 2.4 Staining and confocal microscopy

Myocardial slices were fixed in 4% PFA in PBS immediately after slicing or after 4–6 days in culture and then stained for confocal microscopic imaging as described previously.^43^ The extracellular matrix (ECM) and cell membranes were stained with 40 µg/mL wheat germ agglutinin (WGA) conjugated to AF647 (Thermo Fisher, W32466) in PBS. Nuclei were stained with 2 µg/mL DAPI (Roth, D1306) in PBS. Additionally, as a myocyte marker, α-actinin was stained with a mouse IgG1 primary antibody (Abcam, ab9465) and a goat anti-mouse secondary antibody conjugated to AF488 (Thermo Fisher, A21121). Antibodies were diluted 1:200 and 1:400, respectively, in PBS supplemented with 0.25% Triton-X, 1% bovine serum albumin (BSA), and 5% normal goat serum. Incubation times were at least 6–8 h. After staining, tissues were mounted on a glass slide in Fluoromount G (Sigma, F4680), covered with a coverslip #1.5, and dried for at least 48 h at 40–45% relative humidity. Stained slices were imaged on a Zeiss LSM780 or a Leica STELLARIS confocal microscope with a ×63 oil-immersion lens. From each tissue slice, three 3D stacks of randomly chosen regions were imaged with image dimensions 1280 × 1280 × 125 voxels of 0.1 × 0.1 × 0.2 µm^3^ size. Additionally, from each slice, a 2D confocal scan covering nearly 1.5 mm^2^ was imaged, using the tile scan mode with subsequent stitching.

### 2.5 Image analysis

Confocal images were deconvolved using measured point spread functions. Subsequently, images were segmented and classified to obtain the voxels (3D stacks) or pixels (2D scans) within the images belonging to the ECM and cell membranes, the nuclei, and cardiomyocytes. As described previously,^43,45–47^ this was achieved by histogram-based thresholding, morphological image operators, and watershed-based creation of ECM-enclosed segments. The α-actinin signal was used to extract segments belonging to cardiomyocytes and to subsequently separate the transverse tubular system (t-system) from the surface membranes and the ECM. Cardiomyocyte width was assessed by the minor axes of ellipsoids fitted to segmented cardiomyocytes in 2D scans. t-system density was assessed in 3D image stacks by calculating the intracellular distance of each voxel to its closest t-tubule. Thus, large distances indicate low t-system density and vice versa. The mean t-system distance of three 3D images per tissue slice was averaged and used as one data point for overall comparison and the standard deviation from myocyte to myocyte of each slice to analyze slice inhomogeneity.

### 2.6 X-ray diffraction

Small-angle X-ray scattering (SAXS) measurements were performed with a laboratory X-ray SAXS setup^48^ (Xeuss 2.0, Xenocs, Grenoble, France), equipped with a microfocus sealed tube source (cooper target) and a photon counting pixel detector (Pilatus3R 1M, 981 × 1043 pixels, pixel size 172 × 172 μm, Dectris Ltd, Baden, Switzerland). Subsequently, the samples were washed in PBS and stored at 4°C. For SAXS recordings, the slices were mounted in a sample chamber,^49^ which was built from two polypropylene foils and filled with PBS. The samples were placed in a collimated X-ray beam (0.5 × 0.5 mm^2^) generated by a Genix 3D source (Xenocs) with multilayer optics (adjusted to Cu Kα radiation *E* = 8.04 keV) and scatterless slits. 2D diffraction patterns of the scattered signal were recorded at a distance of 1217 mm. To protect the detector from radiation damage, the primary beam was blocked directly in front of the detector by a beamstop (3 mm). For each sample, four neighbouring positions with a distance of 1 mm were recorded for 1 h (divided into 10 min intervals). The background signal of the chamber and buffer (PBS) was recorded at two positions void of the sample. In order to correct for small variations in sample thickness, the transmission was measured by recording the unblocked direct beam without a sample in the pathway and for each sample position for 0.1 s. For SAXS data analysis, the custom-made nanodiffraction toolbox^49^ implemented in MATLAB (MathWorks, USA) was used. First, the diffraction patterns of each sample position were averaged, and the background was subtracted. The corrected data were angularly averaged to obtain 1D intensity profiles *I*(*q*), which were fitted by a model function.^50^


$$I(q)=S\;q^{-d}+I_{p}\exp(-(q-q_{0}{)}^{2}/\sigma^{2})+I_{\mathrm{bgr}},$$

where *I*_bgr_ denotes the uniform background intensity, the parameter *S* the prefactor and *d* the decay exponent of the power law. Further, a Gaussian (with width *σ*, peak intensity *I*_p_, and lateral peak position *q*_0_) accounts for the characteristic peaks of the actomyosin signal. The interfilament distance *d*(1,0) = 2*π*/*q*_0_ was obtained from lateral peak position *q*_0_ = *q*_(10)_, and the actomyosin lattice spacing *a* is defined by *a* = 4*π*/sqrt(3)/*q*_0_.

### 2.7 Statistics

Dots represent the individual results from one slice (number of individual slices defined by *n* and number of pigs reported as *N*), and aggregated date are given as mean ± standard error of the mean. Statistical analyses were performed with GraphPad Prism 8.0 software. Statistical analysis was performed with the one-way anaysis of variance (ANOVA) test with a Tukey’s multiple comparison test for the comparison of the FFR and contraction duration–frequency ratio changing during long-term cultivation, as well as the changes in ERP after adding ranolazine. A two-way ANOVA and Sidak’s multiple comparison test to test the effect of JNJ 303 in ERP. The proportion of ECM, cardiomyocyte width, t-tubule distance, and standard deviation of mean myocyte t-tubule distance within each slice were analysed with two-tailed unpaired Student’s *t*-tests. We used Kaplan–Meier curves to display the number of slices that were able to generate contraction force above 1 mN during cultivation and the pacing capability. For statistical comparisons of the pacing capability, we used two-sided Fisher’s exact test with time-matched controls. For the FFR, contraction duration–frequency ratio, and Δ baseline, the absolute values were taken into account. For all other parameters, we normalized the absolute values to timepoint 0 of each slice to reduce the variability. Statistical analyses of drug effects were then performed by a two-way ANOVA with Sidak’s multiple comparison test with time-matched controls. To detect arrhythmogenic drugs within the CiPA drug screen, we used a two-way ANOVA with Dunnett’s multiple comparison test comparing all drugs as well as DMSO to the PBS group as control for the respective timepoints.

The EC_50_/IC_50_ values were calculated individually for each slice by the non-linear regression, log (agonist/inhibitor) vs. response function. *P* < 0.05 was considered statistically significant, and significances are indicated as *P < 0.05, **P < 0.01, ***P < 0.001, and ****P < 0.0001.

## 3. Results

**Figure 1:**
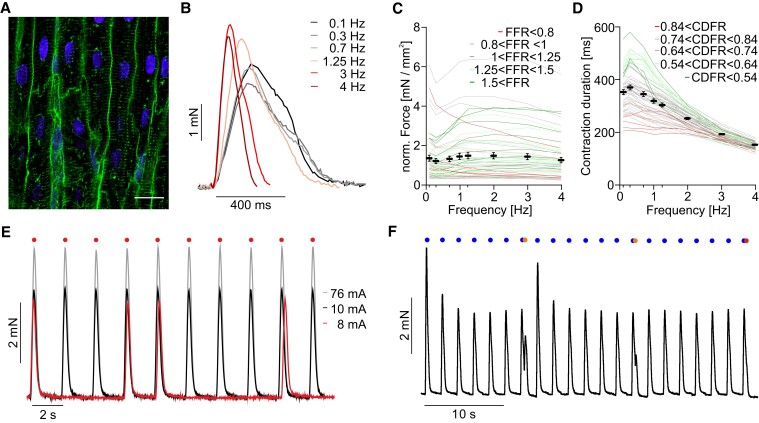
Acute properties of pig ventricular slices. (*A*) Confocal image of a ventricular slice directly after cutting. Nuclei shown in blue, WGA in green, and bar equals 20 µm. (*B–D*) Representative twitch contractions (*B*) and force amplitudes of individual slices (red, grey, and green) and aggregated data (*C*, black, *N* = 5, *n* = 59) as well as individual (red, grey, and green) and mean contraction duration (*D*, black, *N* = 5, *n* = 59) during electrical stimulation with the indicated frequencies. (*E*) Representative force traces of pacing at 0.5 Hz with decreasing electrical current amplitude (3 ms biphasic, 0.5 Hz). (*F*) Representative force traces for analysis of ERP with a S1S2 protocol. Note the failure of the S2 stimulus in the end. S1 stimuli are depicted as blue dots and S2 stimuli as red dots.

We cut 300 µm thick slices and spanned the ones with homogenous and parallel cardiomyocyte alignment selected by visual inspection into BMCS. The cellular structure was confirmed by confocal microscopy. On the molecular level, the actin and myosin arrangement of the slices was probed by X-ray diffraction, which is an additional approach to characterize myopathies.^51^ We determined an interfilament distance of 48 ± 1.9 nm. The cardiomyocyte width was 24 ± 1.5 µm (*N* = 3, *n* = 7), and the average t-tubule distance was 0.59 ± 0.035 µm (*N* = 4, *n* = 8), analysed with confocal imaging (*Figure 1A*). Slices were kept under continuous stimulation with 0.5 Hz and developed 1.3 ± 0.14 mN/mm^2^ at this frequency. Importantly, 78% (*n* = 59) of slices had a positive FFR, which was on average of all slices 1.2 ± 0.03 in ratio (*Figure 1B* and *C*) that is more positive compared to human slices generated from failing hearts.^43^ The shortening of contraction duration with increasing beating rate was even more pronounced with a ratio of 0.69 ± 0.01 (*Figure 1B* and *D*). All slices could be paced with electrical field stimulation (3 ms, biphasic stimulation at 0.5 Hz) with an average pacing threshold of 18 ± 0.87 mA (*Figure 1E*, *n* = 76, *N* = 5). Furthermore, ERP was 420 ± 8.0 ms (*Figure 1F*, *n* = 75, *N* = 5).

**Figure 2:**
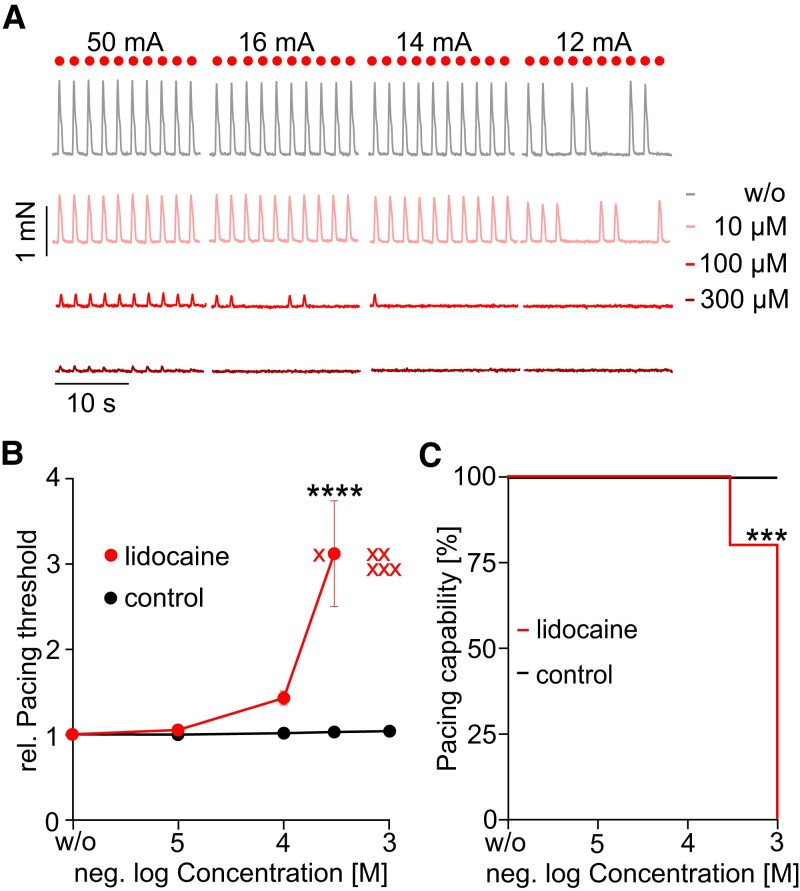
Effects of lidocaine (red, *N* = 2, *n* = 5), a specific blocker of Na_v_1.5 channels. (*A*) Representative twitch contractions before and after adding the indicated concentrations. (*B*) Aggregated data of the normalized pacing threshold in dependence of the concentration (red) compared to time-matched controls (black, *N* = 3, *n* = 8) with a two-way ANOVA with Sidak’s multiple comparison test. Slices, which could not be paced with the highest possible current (∼100 mA), are marked as red X. (*C*) Kaplan–Meier curve displaying the drop in percentage of slices that could be paced by electrical stimulation after applying the different concentrations (red) compared to time-matched controls (black, *N* = 3, *n* = 9) by a two-sided Fisher’s exact test. Exact *n* numbers and *P* values for each condition are given in Supplementary material online, *Table S1*, sheets I and II.

Na_v_1.5 are the major channels contributing to the fast upstroke in the initial phase 0 of the cardiac AP, and, thus, their activation is crucial for recruiting single cardiomyocytes and in consequence conduction of the electrical activation throughout the working myocardium.^52^ Genetic loss-of-function mutations or drug-induced inhibition of the fast initial current of Na_v_1.5 channels lead therefore to the arrhythmogenic Brugada syndrome.^53^ To test how the inhibition of fast Na^+^ currents affects myocardial slice function and how this is reflected in the functional parameters, we applied the specific blocker lidocaine at increasing concentrations. We observed an increase of the pacing threshold starting at 100 µM. From 300 µM on, pacing became inefficient (*Figure 2*; Supplementary material online, *Table S1*, sheet II). Thus, we were not able to determine the IC_50_, but the concentrations causing effects are in the range of reported IC_50_ values from 20 to 200 µM for inhibition of Na_v_1.5 channels, investigated by voltage-clamp patch-clamp measurement of COS-7, hiPSC-CMs,^16^ HEK293, and canine cardiac Purkinje cells.^54^ We also observed a significant decrease in the force, ERP, and contraction duration (see Supplementary material online, *Table S1*, sheet II).

**Figure 3:**
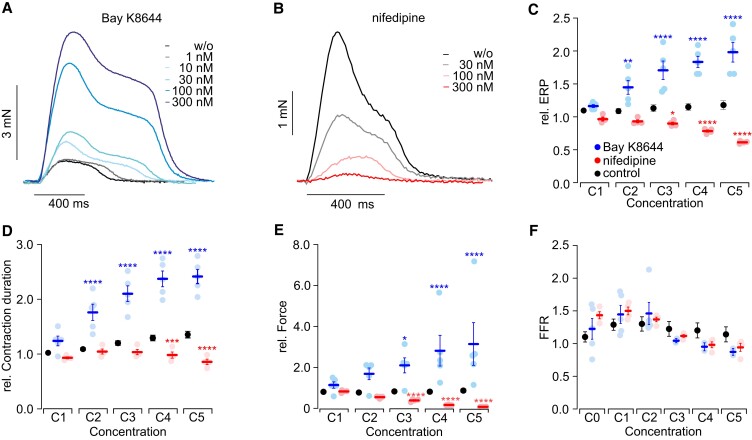
Effects of Bay K8644 (blue, *N* = 2, *n* = 5) and nifedipine (red, *N* = 1, *n* = 4), specific activator and blocker of Ca_v_1.2 channels, respectively. (*A*, *B*) Representative force traces at 0.7 Hz pacing after applying the indicated concentrations of the L-type Ca^2+^ channel activator Bay K8644 (*A*) and blocker nifedipine (*B*). (*C–F*) Aggregated data of the influence of different concentrations on the normalized ERP (*C*), normalized contraction duration (*D*), normalized force at 0.7 Hz pacing (*E*), and the FFR (*F*) of Bay K8644 and nifedipine. Statistical comparison by a two-way ANOVA with Sidak’s multiple comparison test with time-matched controls (black, *N* = 3, *n* = 9). Exact *N* values and *P* values for each condition are given in Supplementary material online, *Table S1*, sheets I, III, and IV.

Ca^2+^ entry through Ca_v_1.2 channels during the plateau and Phase 2 of the cardiac AP is essential to initiate the Ca^2+^-induced Ca^2+^ release in the dyadic cleft, which is the first step of the excitation–contraction coupling and force generation. On the other hand, both activation or gain of function and inhibition or genetic loss of function can be proarrhythmic leading to the long QT or short QT syndrome, respectively. Thus, we tested the selective Ca_v_1.2 activator Bay K8644 (*Figure 3A*) and the inhibitor nifedipine (*Figure 3B*). We found opposing effects in the ERP (*Figure 3C*), contraction duration (*Figure 3D*), and force generation (*Figure 3E*) but not in the FFR (*Figure 3F*). The force increase was induced by Bay K8644 with an EC_50_ of 27 ± 12 nM (*N* = 3, *n* = 7), which is well in line with a report using papillary muscle of human failing hearts.^55^ Furthermore, the EC_50_ of ERP prolongation with Bay K8644 (24 ± 7.1 nM, *N* = 3, *n* = 7) was even lower compared to the EC_50_ determined by voltage imaging of hiPSC-CMs (∼70 nM)^56^ and in the same range reported for voltage-clamp experiments with ventricular cardiomyocytes from guinea pigs.^57^ Also the IC_50_ of nifedipine on contraction force (25 ± 10 nM, *N* = 3, *n* = 9) is in a similar range as in voltage-clamp experiments analysing *I*_Ca_ in HEK293 cells,^58^ CHO cells,^59^ and hiPSC-CMs.^60^

**Figure 4:**
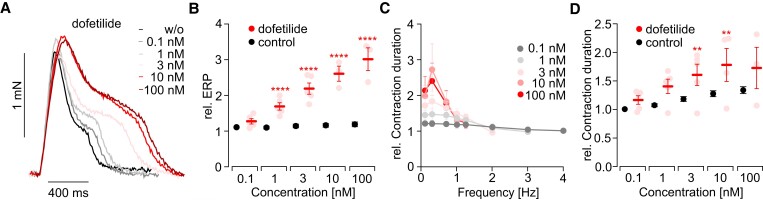
Effects of dofetilide (red, *N* = 2, *n* = 5), a specific blocker of hERG K^+^ channels. (*A*) Representative twitch contractions at 0.7 Hz electrical pacing after applying the indicated concentrations. (*B–D*) Aggregated data showing the effects of dofetilide on the normalized ERP (*B*) and the normalized contraction duration at different pacing frequencies (*C*) and at 0.7 Hz in dependence of the applied concentration (*D*). Exact *N* and *P* values for each condition are given in Supplementary material online, *Table S1*, sheets I and V. Statistical comparison by a two-way ANOVA with Sidak’s multiple comparison test with time-matched controls (black, *N* = 3, *n* = 9).

In Phase 3 of the cardiac AP, voltage-gated K^+^ channels repolarize the membrane towards the resting membrane potential, and the two most important currents are *I*_Kr_ and *I*_Ks_. While genetic mutations leading to loss of function in both channels are the predominant forms of long QT syndrome, the vast majority of drug-induced long QT syndrome can be attributed to the inhibition of *I*_Kr_ because the hERG K^+^ channel cavity is so large that many drugs can enter and alter K^+^ conduction through the pore.^7,61^ We tested dofetilide, a specific blocker of hERG channels (*Figure 4A*), and found a prolongation of the ERP (*Figure 4B*) and contraction duration (*Figure 4C* and *D*) with IC_50_ values of 1.9 ± 0.20 nM (*N* = 2, *n* = 5) and 1.6 ± 0.40 nM (*N* = 2, *n* = 4), respectively, which is even a bit lower compared to recent reports using voltage-clamp experiments in HEK293 cells^62^ and on hiPSC-CMs determined by voltage imaging^63^ as well as the concentrations used in rabbit ventricular cardiomyocytes^62^ and human trabeculae.^64^ Furthermore, we found a much more pronounced prolongation at low beating rates confirming the known rate dependence of *I*_Kr_.^65^

**Figure 5:**
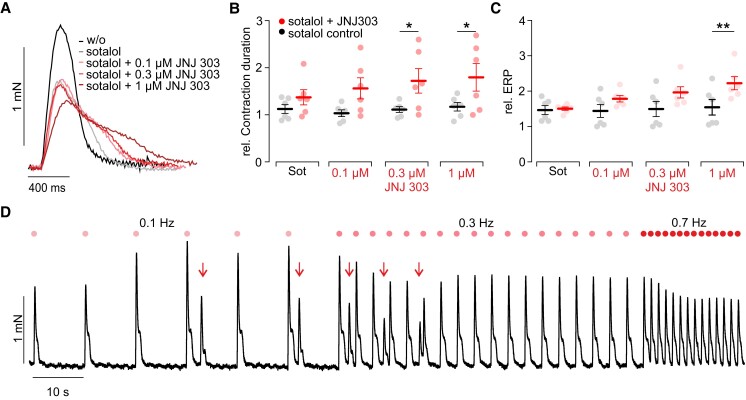
Effects of JNJ 303 (red, *N* = 4, *n* = 6), a specific blocker of KVLQT1 K^+^ channels. (*A*) Representative twitch contractions before and after applying D-sotalol and additionally JNJ 303 at the indicated concentrations at 0.7 Hz pacing rate. (*B*, *C*) Aggregated data of contraction duration at 0.3 Hz pacing rate (*B*) and the ERP (*C*). Statistical comparison by a two-way ANOVA and the Sidak’s multiple comparison test. Exact *N* and *P* values for each condition are given in Supplementary material online, *Table S1*, sheets VIa and VIb. Statistical comparison by a two-way ANOVA with Sidak’s multiple comparison test with time-matched sotalol controls (black, *N* = 2, *n* = 6). (*D*) Force traces with arrhythmic extrabeats (marked by red arrow) after applying 10 µM sotalol and 0.3 µM JNJ 303.

*I*_Kr_ and *I*_Ks_ contribute to the same phase of the AP and are therefore partly redundant building the so-called repolarization reserve. In consequence, potential *I*_Ks_ effects can only be detected after subthreshold blocking of hERG K^+^ channels.^2,66^ Thus, we chose to first apply the hERG and β-adrenergic receptor blocker D-sotalol leading to a slight prolongation of the contraction duration and ERP,^18^ which was significantly more pronounced when adding the *I*_Ks_-specific blocker JNJ 303 at all tested concentrations (*Figure 5A–C*), which are only slightly above the reported IC_50_ values of ∼60 nM.^67^ Importantly, we found in the case of 10 µM sotalol + 0.3 µM JNJ 303 arrhythmic extrabeats between electrically induced contractions (*Figure 5D*), which can be most likely attributed to delayed after depolarizations.^68^ Interestingly, these arrhythmic extrabeats only occurred at lower but not higher beating frequencies.

Glycosides such as digitalis and digitoxin inhibit Na^+^/K^+^ ATPases and have been extensively used to treat heart failure patients. However, their use is nowadays restricted to patients with heart failure and atrial fibrillation with high ventricular beating rates. The initial reason to treat heart failure patients was the positive inotropic effect by intracellular Ca^2+^ accumulation.^69^ Likewise, we were able to detect the positive inotropic effect of ouabain, a specific inhibitor of the Na^+^/K^+^ ATPase, at low concentrations, but we also observed detrimental side effects at slightly higher concentrations, which included contractile oscillations and hypercontraction (see Supplementary material online, *Figure S1A–C*). We observed an inotropic effect starting from 30 nM ouabain. This is well in line with the result from engineered heart muscle that showed a positive inotropic effect after treatment with 100 nM ouabain^26^ and way below experiments with heart tissue from rats.^70^ The increase in diastolic force after 100 nM ouabain is most likely due to the substantial increases in diastolic Ca^2+^ concentrations.^71^ This suggests that with our screening approach, we could also detect cardiotoxicity inducing diastolic dysfunction, as, for example, present in heart failure with preserved ejection fraction. While we did not observe any differences in the contraction duration, the ERP was significantly prolonged, and there was an increase in the pacing threshold at low concentrations (see Supplementary material online, *Figure S1D–F*).

Effects of the sympathetic nervous system on the ventricles during the ‘fight and flight’ response of the body are mediated via β-adrenergic receptor coupling predominantly to the G_s_ protein pathway.^72^ To test the informative value of our approach, we applied isoprenaline, a specific agonist for β-adrenergic receptors (see Supplementary material online, *Figure S2A*). Indeed, we could detect the positive inotropic (see Supplementary material online, *Figure S2A* and *B*), positive bathmotropic effect seen by lower pacing thresholds (see Supplementary material online, *Figure S2C*), positive lusitropic effect (see Supplementary material online, *Figure S2D*) by disinhibition of the SERCA pump, and the shortening of the APD and thus ERP (see Supplementary material online, *Figure S2E*) due to increasing *I*_Ks_ currents. The EC_50_ value for force increase was 69 ± 21 nM (*N* = 3, *n* = 5). Since ventricular slices do not have any spontaneous beating, we were also able to look for the effects at low pacing frequencies and found spontaneous extrabeats occurring at low pacing frequencies, which could be prevented by overpacing with frequencies above 0.7 Hz (see Supplementary material online, *Figure S2F*).

Since many drugs exert not only acute but also or even exclusively long-term effects on heart function, we next explored the feasibility of cultivating the slices with continuous contraction monitoring (see Supplementary material online, *Figure S3A*). Most of the slices showed preserved contractility for 6 days while few were even contracting until Day 23 (see Supplementary material online, *Figure S3B*). During this period, slices first showed a fast increase in force, followed by a slight decrease between Days 2 and 5 (see Supplementary material online, *Figure S3C*). Contraction duration (see Supplementary material online, *Figure S3D*) and ERP (see Supplementary material online, *Figure S3E*), however, were stable until Day 8. The pacing threshold increased slightly during the long-term cultivation (see Supplementary material online, *Figure S3F*). Most myocardial slices showed a reversion of the FFR (see Supplementary material online, *Figure S3G*) during this time period, whereas the CDFR remained stable (see Supplementary material online, *Figure S3H*). This could be a hint that the reversion of FFR from positive to negative can be attributed to a weaker Ca^2+^ channel facilitation by Ca^2+^/calmodulin-mediated modulation of sarcolemmal Ca^2+^ channels^73^ or weaker or less Ca^2+^-induced Ca^2+^ release. Dramatic changes in expression and/or phosphorylation of phospholamban and disinhibition of SERCA pumps are less likely since this should have led to a loss of the frequency-dependent acceleration of relaxation^74^ and thus flattening of the CDFR, which we did not observe (see Supplementary material online, *Figure S3H*).

Histological analysis yielded preserved cardiac structure until Day 6, as indicated by an absence of significant changes in ECM proportion, cardiomyocyte width, and t-tubule distance (see Supplementary material online, *Figure S4A–E*). However, we found an increase in myocyte inhomogeneity revealed by a higher standard deviation of the different regions of interest) analysed within one slice (see Supplementary material online, *Figure S4F*). This could have contributed to the drop in force between Days 2 and 6 (see Supplementary material online, *Figure S3C*) as well as the decrease in FFR happening quickly after the start of incubation (see Supplementary material online, *Figure S3G* and *Table S1*, sheet I) since especially the t-tubule structure and the dyadic cleft are important for efficient excitation–contraction coupling and force generation.^75,76^ Importantly, decreases in t-tubular density, failures in action potential propagations within t-tubules, and detubulation have already been correlated to FFR reversion in single cardiomyocytes^76^ and in slices from failing human hearts. Importantly, the latter displayed much lower FFR even in the best cases compared to the values of fresh slices in this report.^43^

We could for the first time reconstruct the interfilament distance of midmyocardial tissue slices using a laboratory X-ray diffraction setup. Characteristic diffraction signals were detectable throughout d0–d6 (see Supplementary material online, *Figure S4G–I*) indicating highly aligned actomyosin lattice organization even after long-term cultivation. Based on the fits of the angular averaged intensity profiles, we determined a mean interfilament distance of 43 ± 0.6 nm for the incubated slices. Importantly, no significant changes in interfilament distance could be observed during long-term cultivation (see Supplementary material online, *Figure S4J*). These interfilament distances are comparable to previously reported values from human trabeculae^77^ and left ventricular papillary muscles from Yucatan mini-pig hearts.^78^ Confocal microscopy and diffraction analysis confirm structural preservation of the myocardial tissue and will allow thus to detect drug effects on the tissue integrity such as fibrosis or loss of sarcomeres and to directly correlate tissue alignment vs. disarray to the ability of force generation and thus efficiency of the cardiac tissue.^79^

**Figure 6:**
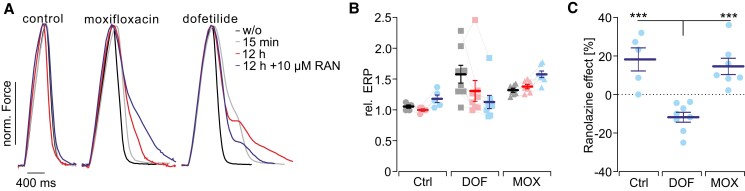
Detection of late Na^+^ currents (late *I*_Na+_). (*A*) Representative force traces before and directly after applying 100 µM moxifloxacin (MOX) or 100 nM dofetilide (DOF), after 12 h incubation and after addition of 10 µM ranolazine (RAN). (*B*) Aggregated data (control: *N* = 3, *n* = 5; DOF: *N* = 5, *n* = 8; MOX: *N* = 4, *n* = 7) of the relative ERP (normalized to before) 15 min after the first treatment (grey), 12 h after the treatment (pink), and after adding 10 µM RAN (blue). (*C*) Changes in ERP after addition of RAN in the respective groups (normalized to 12 h after treatment). Exact *N* and *P* values for each condition are given in Supplementary material online, *Table S1*, sheets IXa, IXb, and IXc. Statistical testing with a one-way ANOVA test and Tukey’s multiple comparison test [*P* (control vs. DOF) = 0.0003; *P* (DOF vs. MOX) = 0.0004; *P* (control vs. MOX) = 0.82].

One cardiac side effect requiring longer time periods is evoking late Na^+^ currents, which has been reported after dofetilide treatment as well as many tyrosine kinase inhibitors used as cancer treatment.^80–82^ We explored overnight incubation with dofetilide to evoke late Na^+^ currents and compared the result to treatment with moxifloxacin, a hERG K^+^ channel blocker without known influence on late Na^+^ currents and control conditions. Twelve hours after the treatment started, we were able to unmask the occurrence of late Na^+^ currents by applying ranolazine (*Figure 6A*). Since ranolazine can inhibit late Na^+^ currents and block hERG K^+^ channels,^83^ it will shorten the AP only if a significant amount of late Na^+^ current is present in cardiomyocytes, such that the AP shortening effect of late Na^+^ inhibition outweighs the AP prolonging effect of *I*_Kr_ inhibition.^84^ As hypothesized, we found ERP shortening after ranolazine only in slices that have been incubated with dofetilide for 12 h but not in controls nor after moxifloxacin treatment (*Figure 6B* and *C*). This provides strong evidence for the feasibility to detect the long-term effects of drugs with our approach.

**Figure 7:**
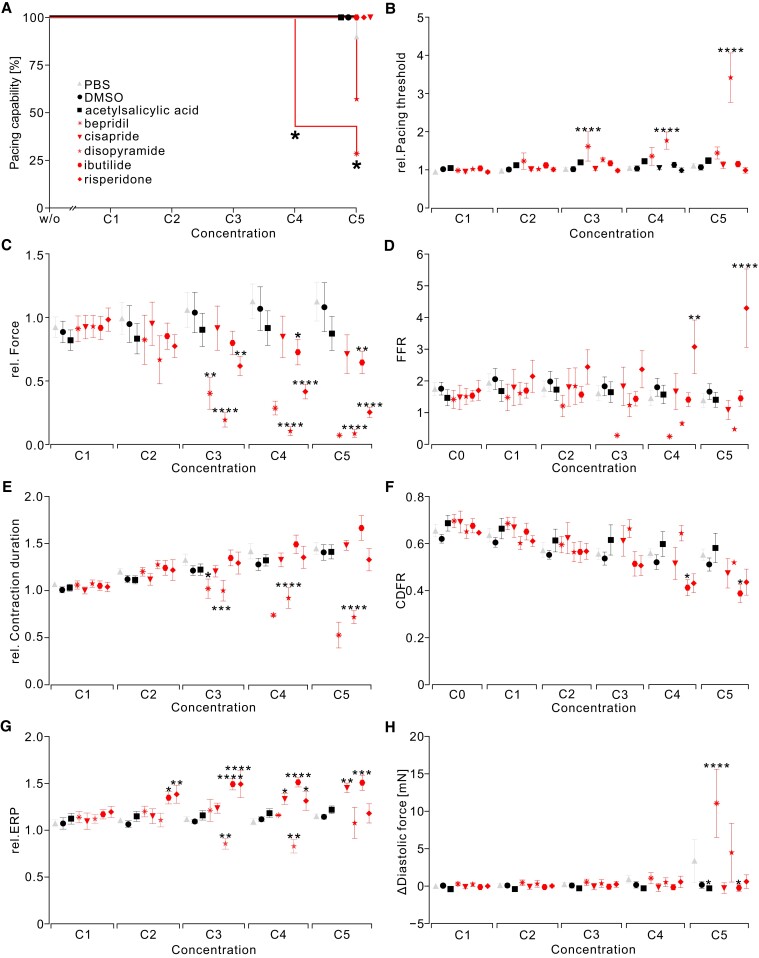
Screening test with drugs from CiPA list with known interactions and risks performed in three different laboratories (*N* = 3–5, *n* = 6–10). The effects of bepridil, cisapride, disopyramide, ibutilide, and risperidone (red) as well as negative controls DMSO and acetylsalicylic acid (black) were compared to time-matched controls treated only with PBS (grey). (*A*) Kaplan–Meier curve displaying the pacing capability of slices. Statistical analysis by Fisher’s exact test. (*B–H*) Mean and SEM of effects compared to PBS with a two-way ANOVA with Dunnett’s multiple comparison test on the normalized pacing threshold (*B*), normalized force (*C*), FFR (*D*), normalized contraction duration (*E*), CDFR (*F*), normalized ERP (*G*), and changes in diastolic force (*H*). Absolute values of each slice were normalized to values before adding the drug or solvent. Exact *n* and *P* values for each condition are given in Supplementary material online, *Table S2*.

Finally, we chose five compounds from the CiPA initiative list^41^ according to the risk stratification list and to cover several possible mechanisms to validate our approach in terms of sensitivity and specificity. We decided to test ibutilide (hERG K^+^ channel blocker, high risk), cisapride (hERG K^+^ channel blocker, intermediate risk), risperidone (hERG K^+^/Na^+^ channel/L-type Ca^2+^ blocker, intermediate risk), bepridil hydrochloride (L-type Ca^2+^/Na^+^ channel/NCX blocker/*I*_Kr_/*I*_Ks_, high risk), and disopyramide phosphate (Na^+^/Ca^2+^/hERG K^+^ channel blocker, high risk) and compared them in a blinded experiment performed by three independent laboratories against PBS and the negative controls DMSO as solvent and acetylsalicylic acid having no known direct side effects on the heart reported (*Figure 7*; Supplementary material online, *Table S2*). Bepridil and disopyramide increased the pacing threshold or even made electrical pacing impossible suggesting inhibitory effects on the fast Na^+^ currents (*Figure 7A* and *B*). These results fit to reports from the literature.^85,86^ We could not detect any effects of risperidone on the pacing threshold. However, risperidone inhibits Na_v_1.6-mediated current, which is one of the major voltage-gated channels in neurons^87^ but does not play a major role in cardiomyocytes.^88^ We also observed the known negative inotropic effect from risperidone, bepridil, and disopyramide (*Figure 7C*). Risperidone decreased the contraction force most likely by blocking the L-type Ca^2+^ channel.^89^ Interestingly, we found that this force decrease is restricted to low frequencies (*Figure 7D*) suggesting a rate-dependent effect of this drug that has not been reported before. Disopyramide has been reported to reduce left ventricular (LV) pressure gradient in patients by affecting Na^+^ and Ca^2+^ channels.^90^ Bepridil exerts significant negative inotropic and chronotropic effects in patients with impaired LV function.^91^ The Ca^2+^ channel–blocking effect of these three negative inotropic substances can be also seen by significant shortening of the contraction duration (*Figure 7E*). Contraction duration, however, was rather insensitive for contraction duration prolongation by the three *I*_Kr_/*I*_Ks_ blockers ibutilide,^92^ cisapride,^93^ and risperidone,^94^ which have been reported to prolong the QT interval of patients. Despite the fact that they all led to a prolongation, this was only in the case of ibutilide significant at 0.3 Hz (see Supplementary material online, *Table S2*, sheet IV), leading also to a significant change in the CDFR (*Figure 7F*), showing the known rate dependence of hERG channels. Importantly, all three led to a significant prolongation of the ERP (*Figure 7G*) but at different concentration levels. This can be explained by counteracting effects on, for example, Ca^2+^ channels kicking in before or after the effect on hERG channels due to different sensitivities. For example, ibutilide led to a significant force reduction at the highest concentration when the ERP prolongation became less pronounced. In the case of bepridil^95^ and disopyramide,^96^ Ca^2+^ channel block was even concealing the ERP prolongation. Bepridil also increased the diastolic force (*Figure 7H*), which further proves that this parameter can be attributed to NCX blockage.^97^ Here, we observed a small but significant decrease induced by acetylsalicylic acid and ibutilide. Importantly, we did not observe any other parameter effects in the control groups, which indicates a relatively high specificity of the presented approach. Furthermore, all drugs from the CiPA list were not only detected but also their characteristic mode of action identified. We were also able to distinguish different targets of one drug by the changes in the respective parameters at increasing concentrations. As an example, bepridil increased the ERP at very low concentrations (0.01–1 μM), which is in agreement with literature values of IC_50_ ∼0.2 μM for blocking hERG K^+^ channels. Ca^2+^ channels are inhibited with an IC_50_ of 0.5 µM.^74^ In well agreement, our experiments showed that 1 µM of bepridil decreased the contraction force to 52 ± 3%. We also observed an increase in the pacing threshold as well as a decrease in pacing capability, but these effects started to occur at higher concentrations (30 µM), which is comparable to the results by patch-clamp measurements analysing Na^+^ channel inhibition (IC_50_: 30 µM).^85^ Bepridil also suppresses *I*_(NCX)_ with an IC_50_ of 8 µM in guinea pigs.^97^ Here, we first saw a relaxation and later an increase in diastolic force at 100 µM (*Figure 7F*).

## 4. Discussion

Herein, we demonstrate and characterize the use of contractility measurements with pig ventricular slices for cardiotoxicity screening to detect changes in electrophysiology as well as contractile behaviour in an unbiased manner. The pig heart is of high relevance for humans due to its comparable size, heart rate, heart rate variability, contractility, and electrophysiology,^98,99^ which is the reason why pig hearts are currently in the focus for xenotransplantation to fight the shortcoming of human hearts as donors for heart transplantation.^100–102^ In regard to electrophysiology, the cardiac transient outward potassium current (*I*_to_) is the only difference that has been reported so far. In humans, *I*_to_ is responsible for the short repolarization during Phase 1 within the cardiac AP. However, the typical notch in Phase 1 can be also observed in pig ventricular cardiomyocytes and has been attributed to Ca^2+^-activated chloride currents (*I*_to2_).^103^ Therefore, one can conclude that the complex activation pattern during the free-running AP is very similar in both species. Accordingly, we were able to demonstrate the effects of specific blockers for all other canonical cardiac ion channels that play an important role in human ventricular cardiomyocytes.^104^ To avoid regional differences affecting the results, we used in this study tissue blocks from the middle of the free left ventricular wall and slices from the midmyocardium of these blocks. Thus, these slices represent the major part of the heart that is most important for its pump function and contains the proposed midmyocardial cells with unusually long APD.^6,99,105^ This might explain why we observed in some cases a prolongating ‘shoulder’ at the end of twitch contractions, especially when reducing the repolarizing K^+^ currents or increasing depolarizing Ca^2+^ currents in case of Bay K8644. This is in line with the effect of isoprenaline, which increases repolarizing *I*_Ks_ currents preventing the occurrence of this phenomenon. Altogether, this could hint at a reduced repolarization reserve in pig ventricular slices, which would explain the high sensitivity for long QT syndrome–inducing drugs and ventricular arrhythmia in pigs.

The results presented here reflect the mean activity of all cardiomyocytes within a slice and thus include the intercell variability of cardiomyocytes. This has recently been shown to be especially important for drug effects.^106^ Our approach will allow to characterize transmural as well as regional differences of the heart. Importantly, one pig heart is still large enough to provide hundreds of such slices for cardiotoxicity screening. Furthermore, the slices comprise all different kinds of cells and structure,^34^ e.g. fibroblasts, endothelial, and smooth muscle cells as well as pericytes, macrophages, and other immune cells that all can influence directly and indirectly cardiomyocytes and the function of the heart.^107–111^

Recording real-time contractions in combination with the presented electrical stimulation protocols enabled us not only to detect drugs affecting cardiac contractility and electrophysiology but also to predict the underlying mechanisms. Affecting Na^+^ channel function can be observed by the pacing threshold, affecting Ca^2+^ channels by the ERP and force amplitude and inhibiting repolarization via *I*_Ks_ and *I*_Kr_ by prolonged ERP. It is important to note that some ion channels can affect several parameters, and it becomes even more complicated if drugs are affecting several ion channels. However, the most important point of cardiotoxicity screening is that drugs influencing the heart function are detected. In the vast majority of our results, the measured EC_50_ and IC_50_ detected were in the same range as in previous reports using predominantly patch-clamp experiments in different cell types including HEK293 cells and hiPSC-CMs and force measurements of trabeculae. The rate control by electrical field stimulation also allows to investigate rate-dependent effects. This is especially important since certain drugs can alter cardiac function only at specific beating rates due to the respective importance of the specific ion channel function as well as use-dependent drug action on ion channels.^68,112–114^ In our experiments, we were able to detect the rate-dependent APD prolongation by *I*_Kr_ blockers in case of ibutilide that is well known as well as the force decrease induced by risperidone only at low beating rates that has not been described yet. Interestingly, we were also able to observe arrhythmia triggers, which can be attributed to delayed after depolarization or early after depolarizations elicited at very long APD after adding sotalol and JNJ 303 (*Figure 5D*) as well as isoprenaline (see Supplementary material online, *Figure S2F*). In both cases, pacing with higher frequencies prevented the occurrence of extrasystolic beats.

It is important to keep in mind that electrophysiological properties and changes were assessed indirectly via the contractile response, and all presented phenomena are indirect predictors of arrhythmia generation that are known to lead to arrhythmia *in vivo* in the intact heart. The main restriction to detect arrhythmia is that the cardiac wavelength^115^ is far too high to fit into the small volume of slices to create self-sustained re-entries and rotors for fibrillating like patterns. Furthermore, the activation pattern induced by field stimulation of a cardiac slice may not replicate the spatiotemporal impulse propagation in the intact heart. This can be, for example, seen by the drop in force when lowering the pacing current (*Figure 1E*) or blocking Na_v_1.5 channels (*Figure 2A*), which can be explained by non-efficient electrical coupling especially in the transversal direction and the known non-homogenous electrical field generation. However, our results strongly indicate that the investigated parameters, comprising pacing threshold, ERP, force amplitude, and contraction duration, are valuable for cardiotoxicity screenings. The important changes can be detected or ideally excluded at first sight in intact tissue without using Langendorff hearts or *in vivo* testing.

As a consequence, we have been able to detect all five compounds taken from the CiPA initiative list in a blinded test screen and could even predict the likely involved ion channels and mechanisms. Importantly, we were also able to detect effects on diastolic tension and provide a pipeline for the analysis of structural changes in cardiac tissue, which is important to predict fibrosis generation and cardiomyocyte survival and thus potential heart failure induction.^1^ In this regard, the ability of long-term cultivation up to 6 days with preserved cardiac structure and contractility is especially intriguing. This enabled us to detect long-term effects of drugs. It has already been demonstrated that cardiac slices from human failing hearts cultivated in BMCS chambers respond to long-term application of pentamidine acting on *I*_Kr_ currents.^38^ Here, using slices from healthy pig hearts, we have been able to evoke late Na^+^ currents by chronic dofetilide exposure. Late Na^+^ currents are recently more and more in focus for cardiotoxicity screening, since some tyrosine kinase inhibitors used in cancer treatment are known to prolong QT duration through this mechanism.^6,80,82^ In addition, stable long-term cultivation for 1 week will allow to explore gene transfer^39,116,117^ and to study potential treatments as well as optogenetic stimulation and imaging to investigate cardiac electrophysiology^118–120^ in intact cardiac tissue without having to perform animal experiments. In the future, systematic media and condition testing as well as cardiac cycle adapted stretch and workload presented recently^121,122^ will further improve functional preservation. The use of optical mapping^117,123^ and multielectrode array recordings^124^ will allow to characterize electrophysiological and electromechanical mechanisms in more detail. In this regard, the precise control of conditions, e.g. media, pre- and afterload, and rate, allows to combine different stress factors such as adrenergic stimulation or hypokalaemia and to look into specific effects like late Na^+^ current generation.^124^

In summary, the ability to screen healthy and intact cardiac tissue with high relevance to humans is a major advantage of this approach since human hearts can only be received for cardiotoxicity screening at a relatively high throughput from heart failure patients in the event of heart transplantation. We suggest that this will improve the predictive value of *in vitro* screening before drugs can be tested in animal models. It will therefore reduce the amount of required animal experiments, withdrawal of falsely classified drugs, and risks of patients in later stages.

## Supplementary material

Supplementary material is available at *Cardiovascular Research* online.

## Authors’ contributions

T.B., Th.S., Ti.S., and A.D. conceptualized the study. M.R., R.S., Ti.S., T.B., A.D. and Th.S. developed the methodology, and R.S. and T.B. wrote the initial draft. R.S., D.J.F., Th.S., A.D., and Z.S. performed the drug screening, and R.S. analysed the drug screening data. M.R., Ti.S., and T.C. performed the X-ray diffraction experiments. L.K.K., D.J.F., and Th.S. acquired and analysed confocal microscopic data.

## Supplementary Material

cvad141_Supplementary_Data

## Data Availability

All data points from BMCS measurements shown in figures for statistical analysis are presented in the Supplementary tables.

## Notes

Translational perspective Cardiotoxicity screening is crucial to lower the risk for patients and avoid economic losses. We demonstrate the value of our approach that detects effects on electrophysiological properties, contractility, and structural remodelling in an unbiased manner. Pig hearts have a high relevance for humans, and the myocardial slices consist of the typical cell composition and tissue architecture. Even after cultivation for days, cardiomyocytes were in rod shape and had an adult t-tubular system with intact excitation–contraction coupling allowing to investigate long-term effects of drugs and therapeutic approaches. Our approach will thus reduce animal experiments as well as improve the predictive value.
