# Correction to: Contractility measurements for cardiotoxicity screening with ventricular myocardial slices of pigs

**DOI:** 10.1093/cvr/cvad187

**Published:** 2023-12-20

This is a correction to: Runzhu Shi, Marius Reichardt, Dominik J Fiegle, Linda K Küpfer, Titus Czajka, Zhengwu Sun, Tim Salditt, Andreas Dendorfer, Thomas Seidel, Tobias Bruegmann, Contractility measurements for cardiotoxicity screening with ventricular myocardial slices of pigs, *Cardiovascular Research*, Volume 119, Issue 14, October 2023, Pages 2469–2481, https://doi.org/10.1093/cvr/cvad141

In the originally published version of this manuscript, the “Published online date”, was incorrectly stated as 20 October 2023. It should be 2 November 2023.

This error has been corrected online.

