# Supplementary material for: Contractility measurements for cardiotoxicity screening with ventricular myocardial slices of pigs
Source: Cardiovasc Res. 2023 Nov 2;119(14):2469–81. doi: 10.1093/cvr/cvad141 (PMC10651213; doi:10.1093/cvr/cvad141)
Supplement: cvad141_Supplementary_Data [file cvad141_supplementary_data.zip › Supplementary figures Shi et al 2023.pdf]

Supplementary files to:

Contractility measurements for cardiotoxicity screening with ventricular myocardial slices of pigs

Short title: Cardiotoxicity screening with pig heart slices

Runzhu Shi[1,2], Marius Reichardt[1,3], Dominik J. Fiegle[4], Linda K. Küpfer[4], Titus Czajka[3], Zhengwu Sun[5], Tim Salditt[3,6], Andreas Dendorfer[5,7], Thomas Seidel[4], Tobias Bruegmann[1,6,8,#]

1 Institute for Cardiovascular Physiology, University Medical Center Göttingen, Göttingen, Germany
2 International research training group 1816, University Medical Center Göttingen, Göttingen, Germany
3 Institute for X-ray Physics, University of Göttingen, Göttingen, Germany
4 Institute of Cellular and Molecular Physiology, Friedrich-Alexander-University Erlangen-Nürnberg, Germany
5 Walter-Brendel-Centre of Experimental Medicine, Hospital of the University München, München, Germany
6 Cluster of Excellence "Multiscale Bioimaging: from Molecular Machines to Networks of Excitable Cells" (MBExC), University of Göttingen, Germany
7 German Centre of Cardiovascular Research (DZHK), Munich Heart Alliance, Munich, Germany
8 German Center for Cardiovascular Research (DZHK), Partner site Göttingen, Göttingen, Germany

Supplementary Figure 1:

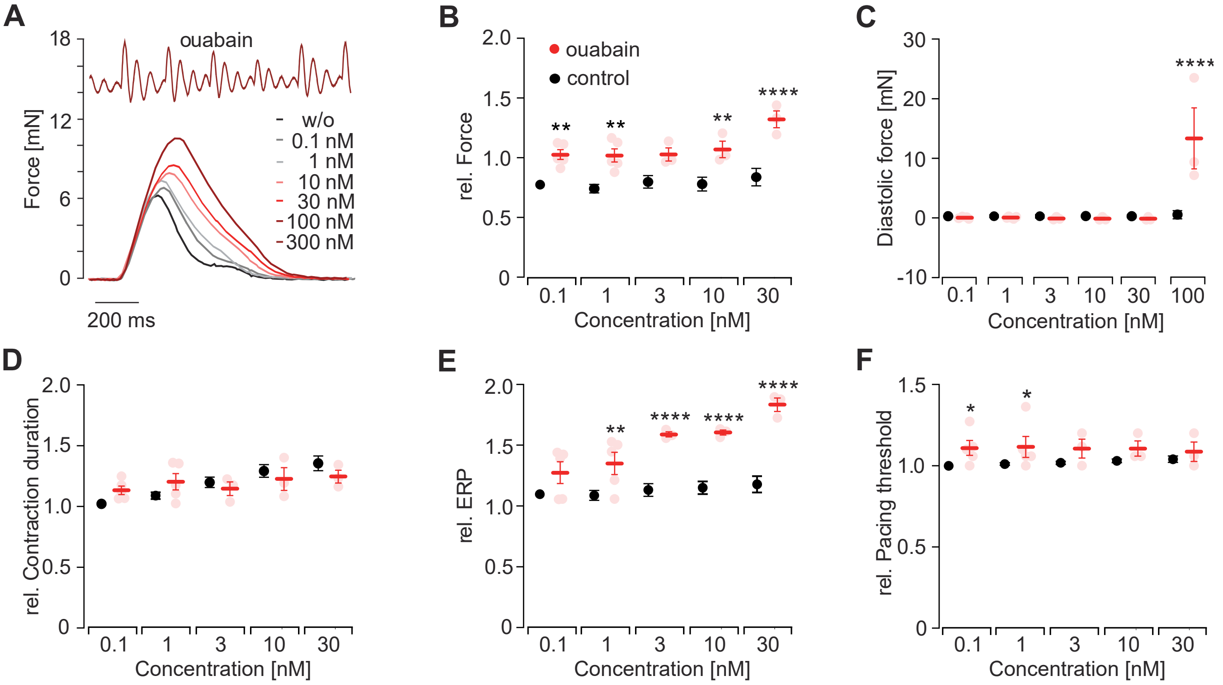

Effects of ouabain (red, N = 2, n = 5), a specific inhibitor of the Na^{+}/K^{+} ATPase. (A) Representative twitch contractions after applying the indicated concentrations of ouabain at 0.7 Hz pacing rate. (B-E) Aggregated data of normalized force (B), the diastolic force (C, note the initial increase in force generation and the later occurrence of hyper-contractions) and contraction duration (D), the normalized ERP (E) and normalized pacing threshold (F). N values and p values are given in Supplementary Table 1, sheet I and VII. Statistical comparison by a two-way ANOVA with Sidak's multiple comparison with time-matched controls (black, N = 3, n = 9).

Supplementary Figure 2:

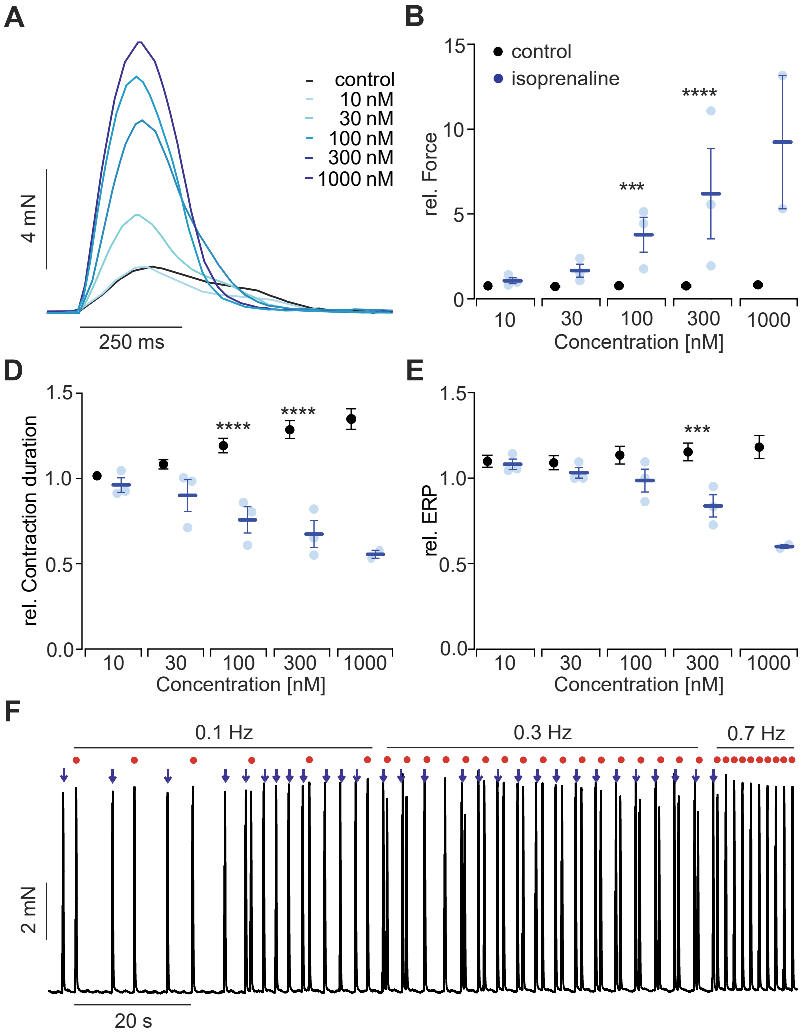

Effects of isoprenaline (blue, N = 2; n = 3), a specific agonist for β-adrenergic-receptors. (A) Representative twitch contractions before and after applying the indicated concentrations. (B – E) Aggregated data of isoprenaline effects on the normalized force (B), normalized pacing threshold (C), normalized contraction duration (D) and normalized ERP (E). Exact N and p values for each condition are given in Supplementary Table 1, sheet I and VIII. Statistical comparison by a two-way ANOVA with Sidak's multiple comparison with time-matched controls (black, N = 3, n = 9). (F) Original force trace after applying 1 µM isoprenaline during electrical pacing (red dots) with 0.1, 0.3 and 0.7 Hz. Arrhythmic extrabeats are marked by blue arrows.

Supplementary Figure 3:

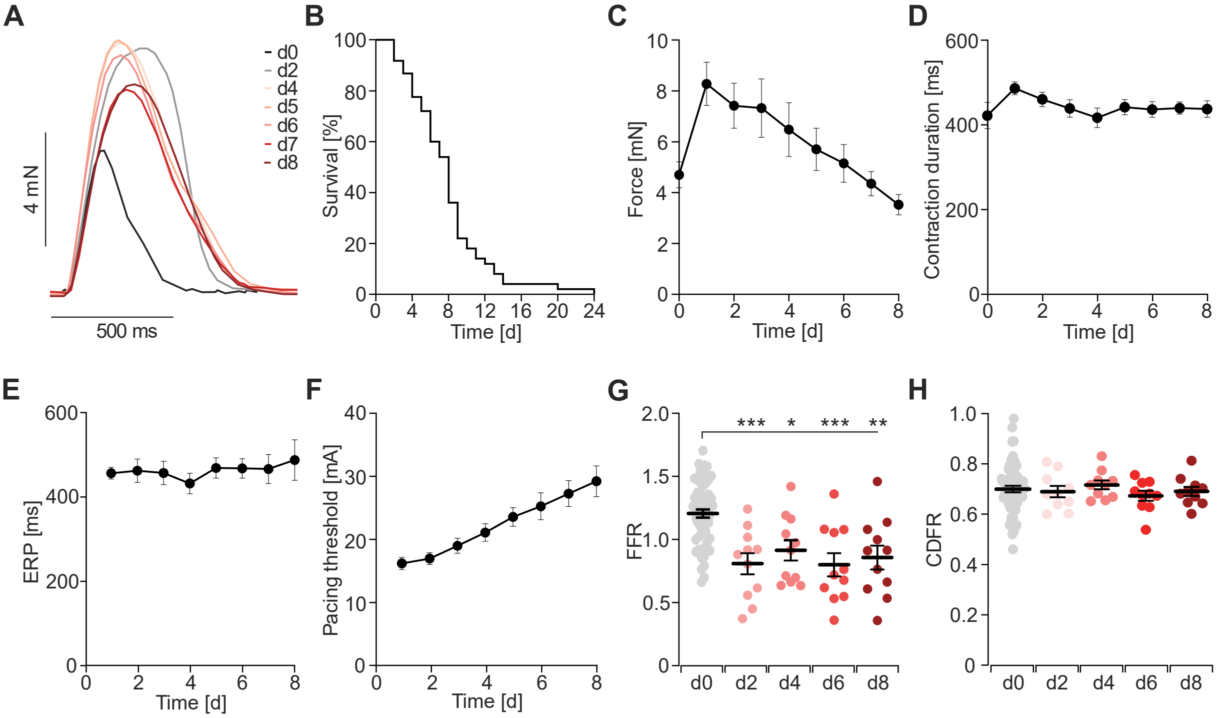

Analysis of contraction parameters during long-term incubation. (A) Representative force traces from d0 - d8 of cultivation and (B) Kaplan-Meier curve displaying the number of slices which were able to generate contraction forces above 1 mN during cultivation (N = 5, n = 61). (C-F) Aggregated data (N = 5, n = 16) of force generation (C), contraction duration (D), ERP (E) and pacing threshold (F) during this period. (G-H) Analysis of FFR (G, force-frequency ratio defined by the force at 1.25 Hz divided by force at 0.3 Hz) and the contraction duration-frequency ratio (H, CDFR defined by the force at 2 Hz divided by force at 0.3 Hz). Statistical testing with a one-way ANOVA test and Tukey's multiple comparisons test. G: p (d0 vs. d2) = 0.0002; p (d0 vs. d4) = 0.012; p (d0 vs. d6) = 0.0001; p (d0 vs. d8) = 0.0014. H: p (d0 vs. d2) > 0.99; p (d0 vs. d4) = 0.98; p (d0 vs. d6) = 0.89; p (d0 vs. d8) > 0.99.

Supplementary Figure 4:

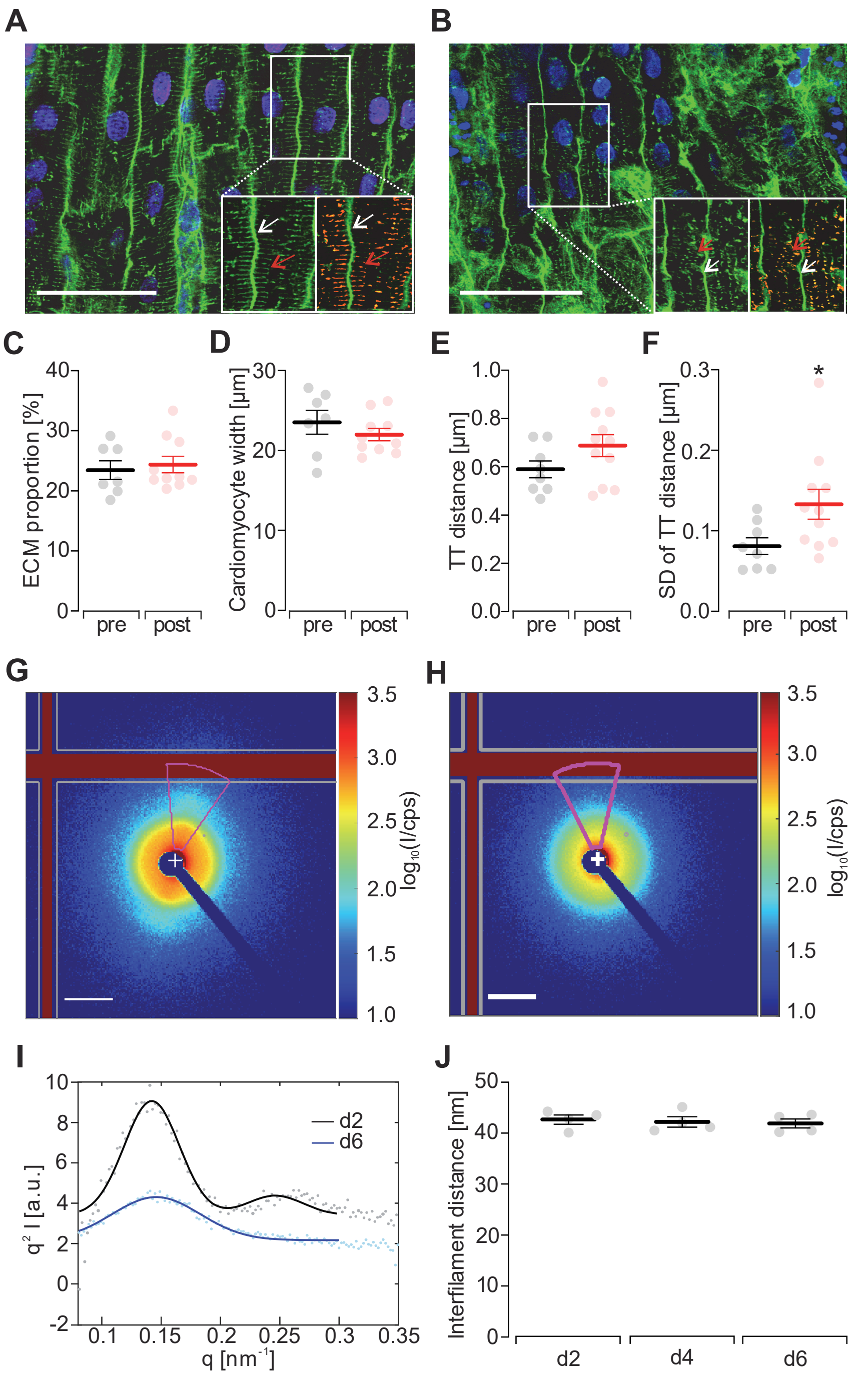

Structure analysis after longterm incubation. (A-B) Representative pictures of myocardial slices pre (A) and 6 days after culture (B), nuclei stained with DAPI (blue), extracellular matrix and membranes stained with wheat germ agglutinin (WGA, green). Bar = 40 µm. Magnified regions show the surface sarcolemma (white arrow) and t-tubules (red arrow, detected by custom-made software). (C-F) Statistical analysis (unpaired student t-test, dots indicate the average result from one slice) of fibrosis (C) indicated by the proportion of extracellular matrix (ECM, p = 0.67, n = 7/10, N = 3/5), of cardiomyocyte width (D, p = 0.33, n = 7/10, N = 3/5) and of t-tubule distance (E, p = 0.13, n = 8/11, N = 4/5) as well as the standard deviation of myocyte t-tubular distance per slice (F, p = 0.04, n = 8/11, N = 4/5). (G-H), Intensity patterns of the X-ray diffraction signal for d2 (G) and d6 (H). The center of the X-ray beam is marked by a white cross and the area of the scattered signal is indicated by the purple mask. Gaps between the detector modules (brown), the beamstop and defect pixels were excluded from the analysis. (I) One-dimensional profile of the scattered intensity (log scale) and the least-square fit along the main scattering angle. The intensity peak around 0.15 nm^{-1} corresponds to the d(0,1) reflection of the actomyosin lattice. (J) Aggregated data of interfilament distance of slices after 2, 4 and 6 days of cultivation.

Supplementary table 1: Excel sheets containing all absolute means and standard error of the means, normalized means and normalized standard error of the means as well as the p values and significances for the time-matched controls (sheet I), lidocaine (sheet II), Bay K8644 (sheet III), nifedipine (sheet IV), dofetilide (sheet V), sotalol only control and sotalol plus JNJ303 (sheet VI a and b), ouabain (sheet VII), isoprenaline (sheet VIII) as well as time-matched controls for moxifloxacine and dofetilide (sheet IX a-c). Statistical comparison test by a two-way ANOVA with Sidak's multiple comparison with time-matched controls and two-sided Fisher's exact test for pacing capability.

Supplementary table 2: Excel sheets indicating the tested drugs, concentrations, the respective pig and slice numbers as well as involved laboratories (sheet I), all absolute means and standard error of the means, normalized means and normalized standard error of the means as well as the p values and significances for the force (sheet II), FFR (sheet III), contraction duration (sheet IV), excitability tests (sheet V), ERP (sheet VI) and diastolic force (sheet VII). Fisher's Exact test was performed for the pacing capability. For the FFR and frequency- contraction duration relation, the statistical analysis was performed with the absolute value using a two-way ANOVA with Dunnett's multiple comparison. For the rest of the parameters, statistical analysis was performed with a two-way ANOVA with Dunnett's multiple comparison with the normalized values with PBS as control group. Only for C0, the absolute values were considered for statistical comparison with one-way ANOVA test and additional Dunnett's multiple comparison test.
